# Supplementary material for: The crystal structure of SUN1-KASH6 reveals an asymmetric LINC complex architecture compatible with nuclear membrane insertion
Source: Commun Biol. 2024 Jan 30;7:138. doi: 10.1038/s42003-024-05794-6 (PMC10827754; doi:10.1038/s42003-024-05794-6)
Supplement: Supplementary file 2 — Supplementary Information [file 42003_2024_5794_MOESM2_ESM.pdf]

Supplementary Information

The crystal structure of SUN1-KASH6 reveals an asymmetric LINC complex architecture compatible with nuclear membrane insertion

Manickam Gurusaran, Benedikte S. Erlandsen and Owen R. Davies

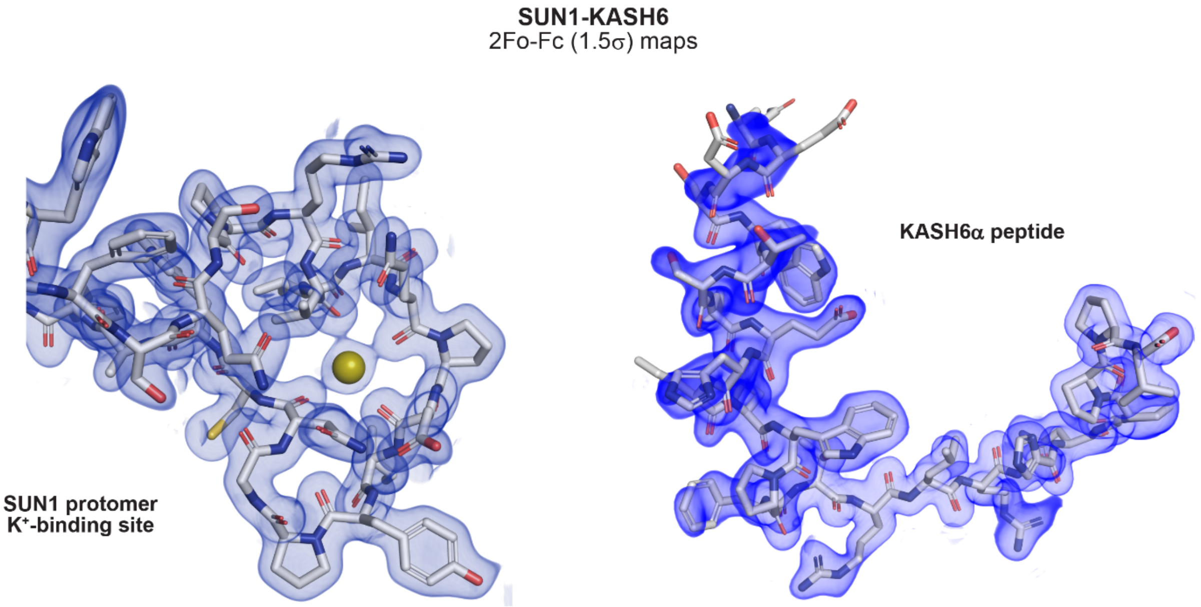

Supplementary Figure 1

Crystal structure of SUN1-KASH6 (1.7 Å) in a 9:9 stoichiometry.

2Fo-Fc electron density maps (1.5σ) of SUN1-KASH6 (1.7 Å) for the potassium-binding site of the SUN1 protomer (left) and the KASH6α peptide (right).

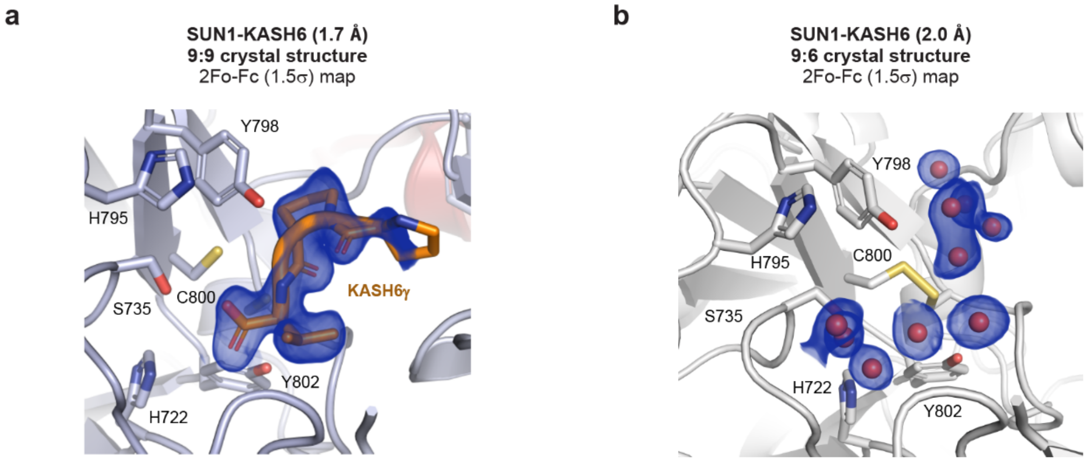

Supplementary Figure 2

Crystal structures of SUN1-KASH6 at the KASH6γ-binding pocket.

(**a,b**) 2Fo-Fc electron density maps (1.5σ) for the KASH6γ peptide and crystallographic water molecules at the KASH6γ-binding pockets of the (**a**) SUN1-KASH6 (1.7 Å) structure at 9:9 stoichiometry, and the (**b**) SUN1-KASH6 (2.0 Å) structure at 9:6 stoichiometry.

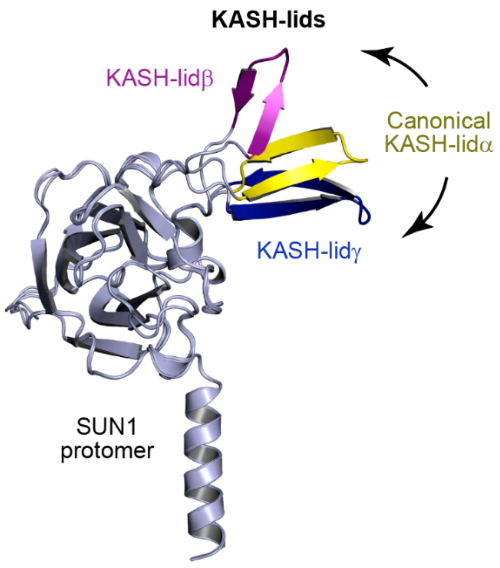

Supplementary Figure 3

Alternative conformations of the three KASH-lids of the SUN1-KASH6 structure.

Superposition of the three SUN1 protomers from one trimer of the structure, showing the canonical orientation of KASH-lidα (yellow), high angulation of KASH-lidβ (purple) and low angulation of KASH-lidγ (blue).

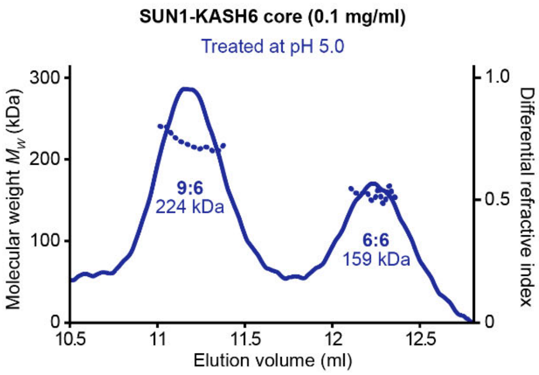

Supplementary Figure 4

SEC-MALS analysis of SUN1-KASH6 core 9:6 and 6:6 species

Size-exclusion chromatography multi-angle light scattering of the SUN1-KASH6 core complex at 0.1 mg/ml following treatment at pH 5.0. This forms 9:6 and 6:6 complexes (224 kDa and 159 kDa; theoretical – 222 kDa and 155 kDa) at approximately 65% and 35% (by mass), respectively. This confirms that 9:6 assembly upon acidic treatment occurs at low protein concentration.

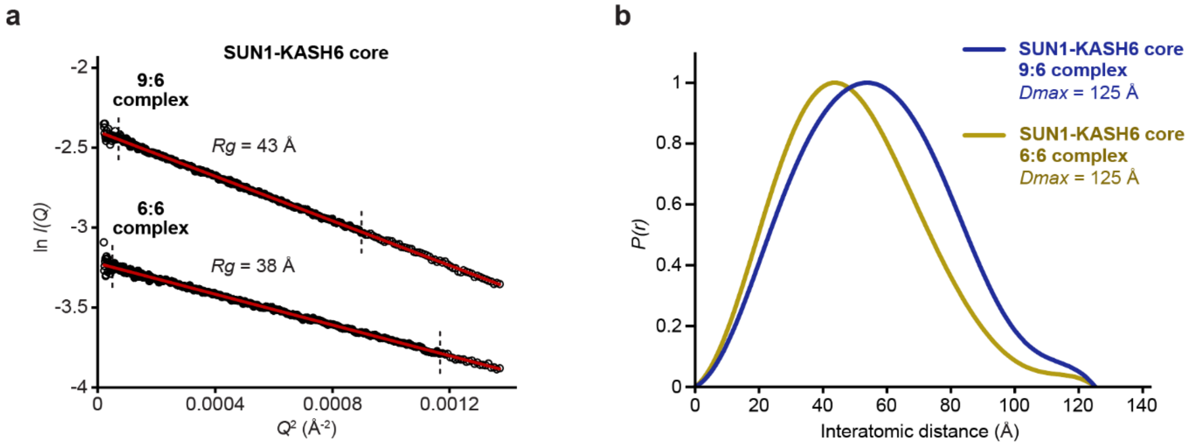

Supplementary Figure 5

Small-angle X-ray scattering (SAXS) of SUN1-KASH6 core 9:6 and 6:6 species

(**a**) SAXS Guinier analysis to determine the radius of gyration (*Rg*); linear fits are shown in red, with the fitted data range highlighted in black and demarcated by dashed lines. The *Q.Rg* values were < 1.3 and *Rg* was calculated as 43 Å and 38 Å for 9:6 and 6:6 species, respectively. (**b**) SAXS *P(r)* interatomic distance distributions in which maximum dimensions (*Dmax*) were determined as 125 Å for both 9:6 and 6:6 species.
