# Supplementary material for: The crystal structure of SUN1-KASH6 reveals an asymmetric LINC complex architecture compatible with nuclear membrane insertion
Source: Commun Biol. 2024 Jan 30;7:138. doi: 10.1038/s42003-024-05794-6 (PMC10827754; doi:10.1038/s42003-024-05794-6)
Supplement: Supplementary file 3 — Description of Additional Supplementary Files [file 42003_2024_5794_MOESM3_ESM.pdf]

Description of Additional Supplementary Files

File name: Supplementary Data 1
Description: Source data for Figure 5c.
